# Supplementary material for: Structural basis of antimicrobial membrane coat assembly by human GBP1
Source: Nat Struct Mol Biol. 2024 Oct 11;32(1):172–84. doi: 10.1038/s41594-024-01400-9 (PMC11746146; doi:10.1038/s41594-024-01400-9)
Supplement: Supplementary file 1 — Supplementary Figs. 1 and 2 and Tables 1–6. [file 41594_2024_1400_MOESM1_ESM.pdf]

nature structural & molecular biology

Supplementary information

https://doi.org/10.1038/s41594-024-01400-9

Structural basis of antimicrobial membrane coat assembly by human GBP1

In the format provided by the authors and unedited

Supplementary Figures, Tables and Information

Structural basis of antimicrobial membrane coat assembly by human GBP1

Tanja Kuhm, Clémence Taisne, Cecilia de Agrela Pinto, Luca Gross, Evdokia A. Giannopoulou, Stefan T. Huber, Els Pardon, Jan Steyaert, Sander J. Tans, and Arjen J. Jakobi

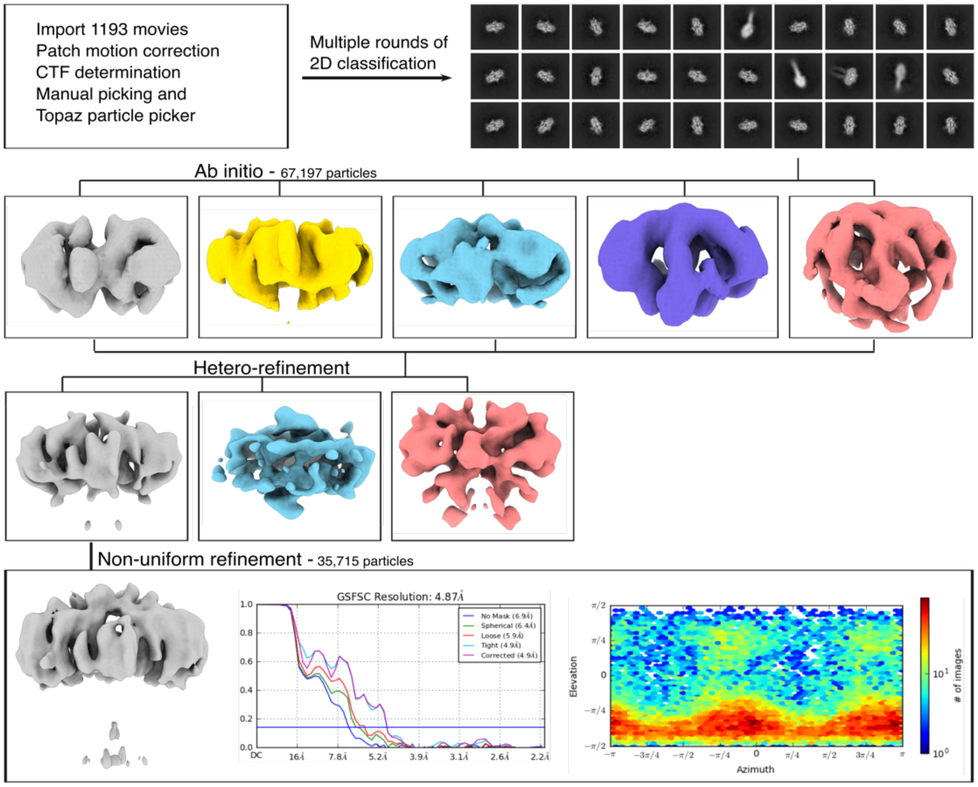

Supplementary Figure 1. Image processing for GBP1-GDP·AlF3. Single-particle analysis processing workflow GBP1-GDP·AlF3 converged on the LG domain dimer. A majority of 2D classes (92% of all particles) showed a top view representative of the LG domain dimer. A subset of 2D classes also comprised the MD domain of both monomers (red boxes).

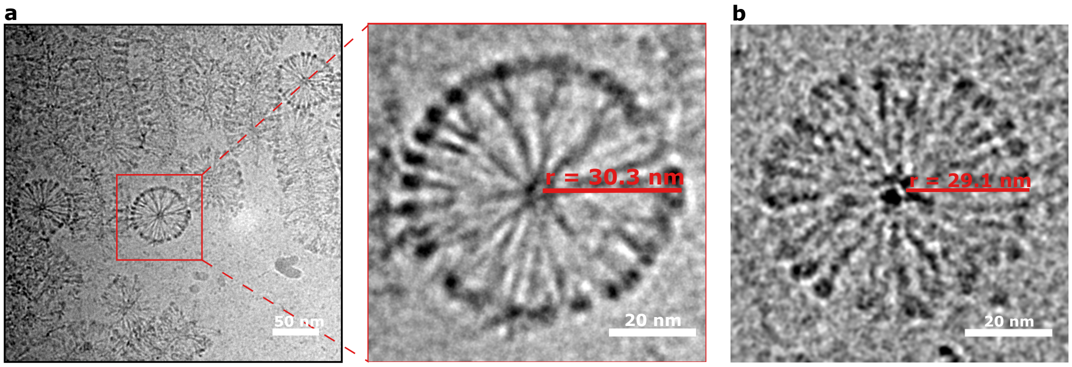

Supplementary Figure 2. Top views of filaments formed by GBP1F on LPS-EB. (a) Putative top vi ews of GBP1F -decorated LPS micelles are indicative of a highly ordered coat. The diameter of these projections was determined to be 60 nm. (b) For comparison, a GBP1F micelle with a diameter of 58 nm is shown.

CA16697 primary sequence

QVQLVESGGGLVQAGDSLRLSCAASGRALGNYYVGWFSQAPGKEREFVAAISWSGSSTFYADSVKGRFTISRDNAKNTVDLQMNSLKPEDT AVYYCAANPTAKLGSSWYQTPREFGYWGQGTQVTVSS

Supplementary Table 1 | Summary of SEC-MALS measurements

	**Average molecular weight [kDa]**	**Number of measurements**
**GBP1**	66.4 ± 2.1 kDa	n = 17
**GBP1-GDP·AlF$_3$**	134.6 kDa ± 4.1 kDa	n = 16
**GBP1$_F$**	66.1 kDa ± 0.3 kDa	n = 7
**GBP1$_F$-GDP·AlF$_3$**	67.6 kDa ± 1.7 kDa	n = 8
**GBP1-Nb74**	79.9 kDa ± 3.0 kDa	n = 2
**GBP1-GDP·AlF$_3$-Nb74**	154.8 kDa ± 4.8 kDa	n = 6
**GBP1$_F$-Nb74**	81.9 kDa	n = 1
**GBP1$_F$-GDP·AlF$_3$-Nb74**	81.9 kDa ± 3.5 kDa	n = 3

* Due to strong preferred orientation, the map resolution range for GBP1-GDP·AlF$_3$ is not conclusive.

Supplementary Table 2 | Summary of data collection and model refinement/validation statistics

	**GBP1-GDP·AlF$_3$**	**GBP1$_F$-GDP·AlF$_3$**
**Data collection**		
Microscope	TFS Titan Krios	JEOL JEM3200FSC
Voltage (kV)	300	300
Detector	Quantum-K2	Quantum-K2
Energy filter	Gatan Bioquantum	In-column omega filter
Micrographs collected (no.)	1193	395 / 606
Pixel size (Å)	1.09	1.22 / 2.449
Electron exposure (e-/Å^2)	60	48 / 13
Frame number	48	60
Exposure time (s)	10	12
Defocus range (µm)	-0.5 to -3.5	-1.0 to -3.0
**Data processing**		
Symmetry imposed	C2	
B-factor (Å^2)	231.1	
Final number of particles	35,715	
Final map resolution (Å)	4.9	
Map resolution range (Å)	*	

* Due to strong preferred orientation, the map resolution range for GBP1-GDP·AlF$_3$ is not conclusive.

Supplementary Table 3 | Summary of data collection parameters of cryo-electron tomogram

	**Tomogram 33 and Tomogram 39**<br><br>**EMD-16813** and **EMD-16814**	**Tomogram 50**<br><br>**EMD-16815**
Content	$GBP1_F$-GDP·AlF_3 + BPLE liposomes	$GBP1_F$-GDP·AlF_3 + BPLE liposomes
Magnification	12,000x	12,000x
Voltage (kV)	300	300
Pixel size (Å)	3.075	3.075
Electron exposure (e-/Å^2)	93.94 (1.54 per increment)	100.04 (1.64 per increment)
Tilt range (°)	-60 to 60	-60 to 60
Increment (°)	2	2
Acquisition scheme	Bidirectional	Bidirectional
Frame number	10 per increment	20 per increment
Exposure time per increment (s)	2.0	3.0
Defocus (µm)	-5.0	-4.0

Supplementary Table 4 | Summary of data collection parameters for LPS datasets

	**LPS-EB**	**LPS-EB-GBP1GDP·AlF_3**	**LPS-SM**	**LPS-SM-$GBP1_F$GDP·AlF_3**	**LPS-ST**	**LPS-ST-$GBP1_F$GDP·AlF_3**
Micrographs collected (no.)	488	561	118	515	79	437
Pixel size (Å)	1.288	1.288	2.449	1.288	1.891	1.891
Electron exposure (e-/Å^2)	46.68	48.01	16.15	43.01	32.02	26.53
Frame number	50	50	50	50	60	48
Exposure time (s)	15	15	15	15	15	12
Defocus range (µm)	-1.0 to -3.5	-1.0 to -3.0	-1.0 to -3.5	-1.0 to -3.5	-1.0 to -3.5	-1.0 to -3.5

Supplementary Table 5 | Vectors and Constructs used in this manuscript.

Name	Description	Source/Reference
AJLV0007	pETM12: *E.coli* expression vector (KanR)	(74)
AJLV0009	pETM14: *E.coli* expression vector (KanR)	(74)
AJLV0038	pCDFDuet: *E.coli* expression vector (SmR)	Merck Millipore (Novagen)
AJLV0040	pJET1.2/blunt: Positive selection cloning vector (AmpR)	Thermo Fisher Scientific
AJLV0068	pcDXNSMG3: FX mammalian expression vector with CMV promoter and N-terminal streptavidin binding peptide, Myc tag, EGFP and 3C protease cleavage site (AmpR)	Addgene 49029
AJLV0081	pINIT: FX sequencing vector (KanR)	Addgene 46973
AJLD0001	Subcloning vector pUC57 with synthetic gene of hGBP1 optimised for *E.coli* expression (AmpR)	GenScript
AJLD0007	GST-tagged-FNTA *in vitro* expression vector pANT7 (AmpR)	DNASU clone HsCD00630808
AJLD0008	GST-tagged-FNTB *in vitro* expression vector pANT7 (AmpR)	DNASU clone HsCD00733069
AJLD0022	NdeI site removed from pANT7-FNTB-cGST (AmpR)	Derived from AJLD0008
AJLD0030	pETM14-hGBP1: *E.coli* expression vector (KanR)	
AJLD0031	pCDF-Duet-FNTA: Intermediate vector for cloning purposes (SmR)	Derived from AJLV0038 and AJLD0007
AJLD0035	pCDFDuet-FNTA-FNTB: *E.coli* co-expression vector for *in vivo* farnesylation of hGBP1 (SmR)	Derived from AJLV0038 and AJLD0022
AJLD0052	pJET1.2 His6-FNTA: Intermediate vector for cloning purposes (AmpR)	Derived from AJLV0040 and AJLD0007
AJLD0053	pJET1.2 FNTB (NdeI removed): Intermediate vector for cloning purposes (AmpR)	Derived from AJLV0040 and AJLD0022
AJLD0056	pETM14-hGBP1-Q577C: *E.coli* expression vector (KanR), point mutation for site-specific labelling	Derived from AJLD0030
AJLD0063	pCDFDuet-His-FNTA-FNTB: *E.coli* co-expression vector (SmR) to express His-FNTA-FNTB	Derived from AJLV0038, AJLD0052 and AJLD0053
AJLD0074	pMES4y-CA16697: C-His6-tagged nanobody (Nb74) raised against farnesylated GBP1	Instruct-ERIC (PID7267)
AJLD0147	pETM14-hGBP1-Y143A: *E.coli* expression vector (KanR) with point mutation	Derived from AJLD0030
AJLD0150	pETM14-hGBP1-L316A-V317A: *E.coli* expression vector (KanR) with point mutation	Derived from AJLD0030
AJLD0151	pETM14-hGBP1-D308S: *E.coli* expression vector (KanR) with point mutation	Derived from AJLD0030
AJLD0153	pETM14-hGBP1-D308A-L309A-P310A: *E.coli* expression vector (KanR) with point mutation	Derived from AJLD0030
AJLD0158	pETM14-hGBP1-K466D: *E.coli* expression vector (KanR) with point mutation	derived from AJLD0030
AJLD0244	pUC57-hGBP1-R370D: Subcloning vector with point mutation	Derived from AJLD0001

AJLD0247	pETM14-hGBP1-R370D: *E.coli* expression vector (KanR) with point mutation	Derived from AJLD0244 and AJLV0009
AJLD0272	pFPV25.1-mCerulean3: Bacterial expression vector (AmpR) with rpsM promoter driving expression of mCerulean3 (codon-optimized for S. typhimurium)	Derived from pFPV25.1-GFPmut3 (Addgene 20668)
AJLD0274	pcDXC3GMSG3-Nb74 : Mammalian expression of GFP-Nb74	Derived from AJLV0068 and AJLD0296
AJLD0276	pLV-Tet-mCherry-hGBP1 WT : plasmid to produce Lentiviral vector LV TRE3G mCherryhGBP1 WT	PolyPlus
AJLD0279	pLV-Tet-mCherry-hGBP1 D308S : plasmid to produce Lentiviral vector LV TRE3G mCherryhGBP1 D	PolyPlus
AJLD0278	pLV-Tet-mCherry-hGBP1 K466D : plasmid to produce Lentiviral vector LV TRE3G mCherryhGBP1 K	PolyPlus
AJLD0280	pLV-Tet-mCherry-hGBP1 Y143A : plasmid to produce Lentiviral vector LV TRE3G mCherryhGBP1 Y	PolyPlus
AJLD0277	pLV-Tet-mCherry-hGBP1 DLP308-310AAA : plasmid to produce Lentiviral vector LV TRE3G mCherryhGBP1 DLP	PolyPlus
AJLD0289	pETM12-GFP-Nb74: Bacterial expression of GFP-Nb74 (KanR)	Derived from AJLV0007 and AJLD0274
AJLD0296	pINIT-kan-GFP-Nb74: Subcloning vector of GFP-Nb74 (KanR)	Derived from AJLV0074 and AJLV0081

Supplementary Table 5 | Bacterial strains and mammalian cell lines used in this manuscript.

**Strain**	**Description**	**Reference**
E.coli DH5α	Cloning host	Invitrogen
E.coli BL21(DE3)	Expression host	Thermo Fisher Scientific (75)
E.coli WK6	Periplasmic expression host	Zell & Fritz, 1987 (76)
HeLa (ACC57)	Human epithelial cell line	DSMZ
HeLa *ΔGBP1* + Tet-mCherry-*GBP1*	CRISPR-engineered cell line	This study
HeLa *ΔGBP1* + Tet-mCherry-*GBP1*-D308S	CRISPR-engineered cell line	This study
HeLa *ΔGBP1* + Tet-mCherry-*GBP1*-D308/L309/P310A	CRISPR-engineered cell line	This study
HeLa *ΔGBP1* + Tet-mCherry-*GBP1*-Y143A	CRISPR-engineered cell line	This study
HeLa *ΔGBP1* + Tet-mCherry-*GBP1*-K466A	CRISPR-engineered cell line	This stuudy
Salmonella enterica subsp. *enterica* serovar Typhimurium	Bacterial Pathogen	DSMZ 19587
