# Supplementary material for: Structural basis of antimicrobial membrane coat assembly by human GBP1
Source: Nat Struct Mol Biol. 2024 Oct 11;32(1):172–84. doi: 10.1038/s41594-024-01400-9 (PMC11746146; doi:10.1038/s41594-024-01400-9)
Supplement: Supplementary file 2 — Reporting Summary [file 41594_2024_1400_MOESM2_ESM.pdf]

nature portfolio

Corresponding author(s): Arjen Jakobi

Last updated by author(s): Mar 19, 2024

Reporting Summary

Nature Portfolio wishes to improve the reproducibility of the work that we publish. This form provides structure for consistency and transparency in reporting. For further information on Nature Portfolio policies, see our Editorial Policies and the Editorial Policy Checklist.

Statistics

For all statistical analyses, confirm that the following items are present in the figure legend, table legend, main text, or Methods section.

n/a	Confirmed	
☐	☒	The exact sample size (*n*) for each experimental group/condition, given as a discrete number and unit of measurement
☐	☒	A statement on whether measurements were taken from distinct samples or whether the same sample was measured repeatedly
☐	☒	The statistical test(s) used AND whether they are one- or two-sided<br>*Only common tests should be described solely by name; describe more complex techniques in the Methods section.*
☒	☐	A description of all covariates tested
☒	☐	A description of any assumptions or corrections, such as tests of normality and adjustment for multiple comparisons
☐	☒	A full description of the statistical parameters including central tendency (e.g. means) or other basic estimates (e.g. regression coefficient) AND variation (e.g. standard deviation) or associated estimates of uncertainty (e.g. confidence intervals)
☐	☒	For null hypothesis testing, the test statistic (e.g. *F*, *t*, *r*) with confidence intervals, effect sizes, degrees of freedom and *P* value noted<br>*Give P values as exact values whenever suitable.*
☒	☐	For Bayesian analysis, information on the choice of priors and Markov chain Monte Carlo settings
☒	☐	For hierarchical and complex designs, identification of the appropriate level for tests and full reporting of outcomes
☒	☐	Estimates of effect sizes (e.g. Cohen's *d*, Pearson's *r*), indicating how they were calculated

Our web collection on statistics for biologists contains articles on many of the points above.

Software and code

Policy information about availability of computer code

Data collection	Serial EM 4.0.0, EPU 2.8.1, AcquireMP software v2.3 and v2.4
Data analysis	ImageJ 2.0.0, cryoSPARC v3.1.0 and v3.3.2, Phenix 1.19, Coot 1.9, LocScale v2.1.2, Refmac5, Servalcat v0.3.0, MotionCor2, IMOD 4.9.2, EMAN 2.3.1, TOPAZ v0.23, ConSurf webserver, MView webserver, HMMer v3.3.2, Image Lab 6.1.0.07, ASTRA v7.3.1, AcquireMP software v2.3 and v2.4. LocScale v2.1.2 is available on https://gitlab.tudelft.nl/aj-lab/locscale.

For manuscripts utilizing custom algorithms or software that are central to the research but not yet described in published literature, software must be made available to editors and reviewers. We strongly encourage code deposition in a community repository (e.g. GitHub). See the Nature Portfolio guidelines for submitting code & software for further information.

Data

Policy information about availability of data

All manuscripts must include a data availability statement. This statement should provide the following information, where applicable:

- Accession codes, unique identifiers, or web links for publicly available datasets
- A description of any restrictions on data availability
- For clinical datasets or third party data, please ensure that the statement adheres to our policy

The refined atomic model of the pseudo-symmetric GBP1 dimer has been deposited in the Protein Data Bank under accession code 8CQB. The primary cryo-EM density and the LocScale map of the pseudo-symmetric GBP1 dimer are available in the E1dg3lectron Microscopy Data Bank (EMDB) under accession code

EMD-16794. Tomogram reconstructions have been deposited at the EMDB under accession codes EMD-16813, EMD-16814 and EMD-16815. Raw micrographs have been deposited in the Electron Microscopy Public Image Archive (EMPIAR) with accession code EMPIAR-11459. Raw tomographic tilt series are available on Zenodo under https://doi.org/10.5281/zenodo.7740464. Atomic model coordinates for PDB IDs 2b92, 1dg3 and 7e5a were retrieved from the Protein Data Bank.

Human research participants

Policy information about studies involving human research participants and Sex and Gender in Research.

Reporting on sex and gender	n/a
Population characteristics	n/a
Recruitment	n/a
Ethics oversight	n/a

Note that full information on the approval of the study protocol must also be provided in the manuscript.

Field-specific reporting

Please select the one below that is the best fit for your research. If you are not sure, read the appropriate sections before making your selection.

☒ Life sciences ☐ Behavioural & social sciences ☐ Ecological, evolutionary & environmental sciences

For a reference copy of the document with all sections, see nature.com/documents/nr-reporting-summary-flat.pdf

Life sciences study design

All studies must disclose on these points even when the disclosure is negative.

Sample size	No statistical method was used to determine sample size. A sample size of n=3 or larger was used for all experiments according to standard practice for data validation and reproducibility
Data exclusions	During the analysis of EM images, micrographs with thick ice or excessive particle motion were excluded. Two-dimension (2D) and three-dimension (3D) classification of the particles was performed to exclude non-specimen related particles. For all other experiments no data was excluded during analysis.
Replication	All independent biological replications were successful and included. Exact numbers are included in figure legends.
Randomization	No experimental groups were present in this study. There was no allocation. Randomization is not relevant in this study.
Blinding	No blinding was required since randomized group allocation was not performed in this study.

Reporting for specific materials, systems and methods

We require information from authors about some types of materials, experimental systems and methods used in many studies. Here, indicate whether each material, system or method listed is relevant to your study. If you are not sure if a list item applies to your research, read the appropriate section before selecting a response.

Materials & experimental systems

n/a	Involved in the study	
☐	☒	Antibodies
☐	☒	Eukaryotic cell lines
☒	☐	Palaeontology and archaeology
☒	☐	Animals and other organisms
☒	☐	Clinical data
☒	☐	Dual use research of concern

Methods

n/a	Involved in the study	
☒	☐	ChIP-seq
☒	☐	Flow cytometry
☒	☐	MRI-based neuroimaging

Antibodies

Antibodies used	rat monoclonal anti-hGBP1 (Santa Cruz Biotechnology; sc-53857); mouse monoclonal anti-c-Myc (MA1-980, Invitrogen); mouse

Antibodies used	monoclonal anti-beta actin (MA1-140, Invitrogen; Goat anti-rat IgG HRP (112-035-003, Jackson ImmunoResearch); Horse anti-mouse-IgG HRP (7076, Cell Signaling Technologies)
Validation	anti-hGBP1: PMID: 18260761, PMID: 23405236<br>anti-c-Myc: This Antibody was verified by Relative expression to ensure that the antibody binds to the antigen stated.

Eukaryotic cell lines

Policy information about cell lines and Sex and Gender in Research

Cell line source(s)	HeLa: DSMZ-German Collection of Microorganisms and Cell Cultures GmbH (ACC 57)<br>HeLa GBP1 KO: CRISPR/Cas9 engineered cell line from the original HeLa source<br>Hela GBP1 KO Tet-mCherry-GBP1: derived from HeLa GBP1 KO by lentiviral transduction<br>Hela GBP1 KO Tet-mCherry-GBP1-D308A/L309A/P310A: derived from HeLa GBP1 KO by lentiviral transduction<br>Hela GBP1 KO Tet-mCherry-GBP1-D308S: derived from HeLa GBP1 KO by lentiviral transduction<br>Hela GBP1 KO Tet-mCherry-GBP1-Y143A: derived from HeLa GBP1 KO by lentiviral transduction<br>Hela GBP1 KO Tet-mCherry-GBP1-K466D: derived from HeLa GBP1 KO by lentiviral transduction
Authentication	Cell lines were not authenticated.
Mycoplasma contamination	All cell lines tested negative for cytoplasma
Commonly misidentified lines<br>(See ICLAC register)	*Name any commonly misidentified cell lines used in the study and provide a rationale for their use.*
