# Supplementary figures and images for: Structural basis of antimicrobial membrane coat assembly by human GBP1
Source: Nat Struct Mol Biol. 2024 Oct 11;32(1):172–84. doi: 10.1038/s41594-024-01400-9 (PMC11746146; doi:10.1038/s41594-024-01400-9)

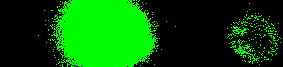

Supplement: Supplementary file 6 — Confocal imaging of GBP1F -dependent membrane fragmentation and lipid transfer. [file 41594_2024_1400_MOESM6_ESM.gif]

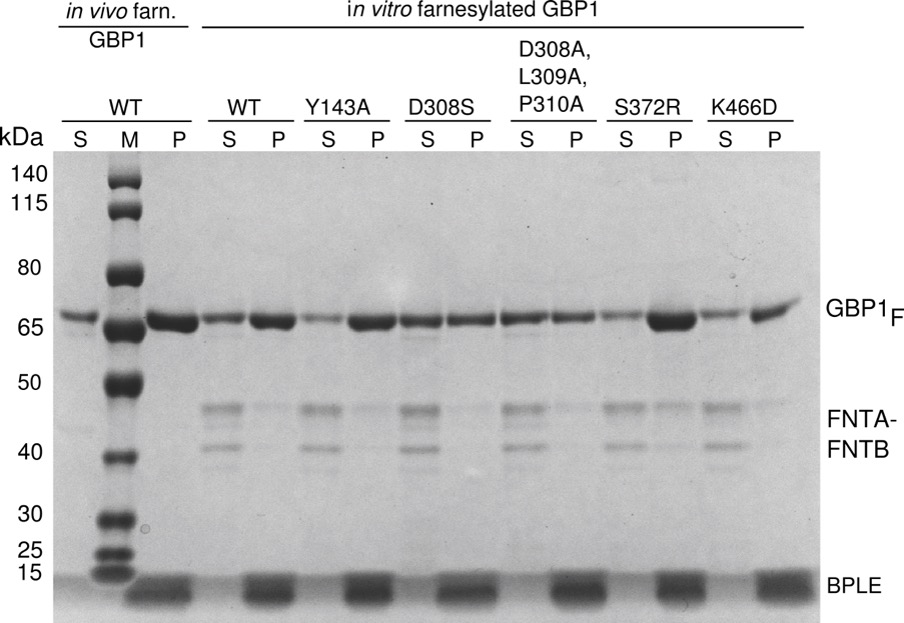

Supplement: Supplementary file 13 — Uncropped gel. [file 41594_2024_1400_MOESM13_ESM.jpg]

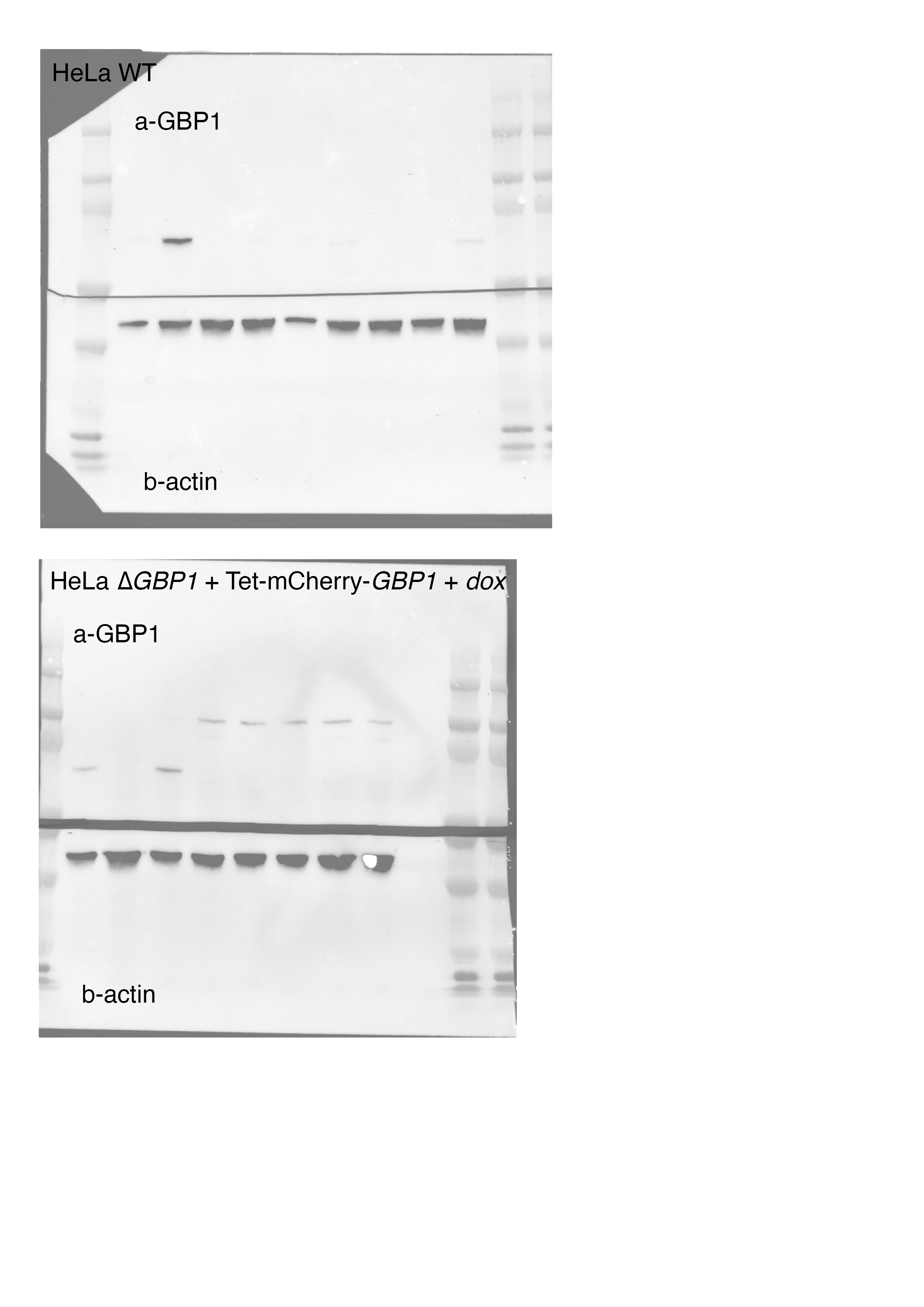

Supplement: Supplementary file 15 — Uncropped western blots. [file 41594_2024_1400_MOESM15_ESM.jpg]
